# Supermarket interventions to improve dietary and lifestyle behaviours: what is the key to success?

**DOI:** 10.1186/s12916-024-03306-1

**Published:** 2024-02-27

**Authors:** Carmen Piernas

**Affiliations:** Department of Biochemistry and Molecular Biology II, Faculty of Pharmacy, Centre for Biomedical Research, Institute of Nutrition and Food Technology, Biosanitary Research Institute, University of Granada, Granada, Spain

**Keywords:** Supermarket, Intervention, Food choices, Non-communicable disease

## Background

There is an urgent need to tackle poor diets and lack of physical activity, both of which are major contributors to the burden of non-communicable disease globally [1]. Over the past few decades, interest has been shifting from individual-level to environmental-level interventions to help change and sustain behaviour and improve health outcomes. Supermarket environments have gained particular attention, given that in many countries across the world, grocery expenditure contributes a high percentage of the total food consumption, constituting an ideal setting to shape food choice.

Previous studies conducted in real-world supermarkets have identified strategies showing various levels of success to support dietary change. The strongest evidence of effect has been reported for economic interventions, namely pricing and promotional strategies that alter the price of a product through discounts or multi-buy offers among others [2, 3]. Other examples of so-called nudging interventions, such as positioning of products in prominent aisles or high-traffic areas [3–5]; and availability interventions altering the amount or proportion of a product on sale have also shown some evidence of effectiveness [4]. Strategies solely based on educating customers through signage, flyers or shelf tags have not shown very significant benefits [3]. Collectively, evidence shows that the impact of single interventions is usually small, mostly focused on increasing sales of healthier foods for short-term periods and rarely applicable to broader food categories, particularly those concerning health the most.

## Real-world nudging and pricing interventions: do they work?

A recent study by Stuber and colleagues in BMC Medicine reports the results of a real-world nudging and pricing intervention within supermarkets, coupled with a mobile physical activity coaching for individuals to improve lifestyle behaviours and cardiometabolic health [6] The Supreme Nudge study is a parallel cluster-randomised controlled trial including 12 supermarkets located in lower sociodemographic neighbourhoods across the Netherlands. Supermarkets were randomised to carry their business as usual (control) or to implement a series of co-designed interventions to promote healthier purchasing. A sample of 361 individuals within those supermarkets were then randomised to receive physical activity coaching through a mobile app or a simpler step counter app. Participants were followed for up to 12 months to see if there were improvements in dietary quality, food purchasing behaviours, physical activity and cardiometabolic risk factors; however, the authors reported that none of these outcomes changed significantly compared to the control group at the end of the study.

The Supreme Nudge study is an example of a carefully designed complex intervention targeting individuals to boost motivation to increase physical activity, as well as the environment to promote healthier food choices. However, the lack of effectiveness across all outcomes deserves some consideration to understand the reasons for that. The supermarket intervention was mostly focused on nudging strategies targeting healthier foods, including product shelf location, availability and signage for about 9% of all products sold in the supermarket. Pricing strategies consisted of price reductions of healthier products and price increases of unhealthier products, targeting about 3% of all products and rolled out for short time periods within each food category. As with other lower-risk supermarket-led interventions that reach a compromise between effectiveness, sustainability and feasibility, the impact is too small to be detected. However, there are examples of co-designed interventions with supermarkets that have provided evidence of improved shopping behaviours without affecting business negatively. Successful interventions have specifically relied on restricting price promotions, positioning and availability across a whole range of discretionary foods and beverages [7, 8], rather than focusing primarily or exclusively on encouraging healthier choices through a single intervention mode.

One important difference between the Supreme Nudge study and others is the use of individual dietary intake measures as primary outcome for up to 12 months. A large body of evidence has reported short-term impact in terms of sales of targeted products, without considering how other products outside of the target categories are marketed or any compensatory effects. Therefore, changes in sales or purchasing behaviour may not necessarily translate into individual changes of sufficient magnitude to improve dietary quality in the long term. Although Stuber and colleagues observed no significant changes in terms of dietary intake or food shopping behaviour, the use of food frequency questionnaires, which are prone to reporting biases and less sensitive for detecting changes over time, could have contributed to the lack of results. Finally, the Supreme Nudge trial was conducted during the COVID-19 pandemic, which was reflected by major changes to their initial protocol and could have particularly affected the typical customer behaviour due to the imposed restrictions.

## Conclusions

Governments are increasingly demanding action from the food industry to support healthier choices, investors are looking for responsible business practices, and consumers are showing a greater interest in healthier options. Within supermarkets, the range of products sold and the ways in which they are priced, placed and promoted is one of the biggest influences on consumer diets. Impactful, scalable and lasting approaches are particularly needed in order to shift population-level intakes to be closer to dietary recommendations. Achieving this will likely require an integrated strategy using multiple interventions simultaneously targeting entire food categories as well as across different categories, whilst considering all aspects of a store operation. Supermarket interventions, if planned with a clear purpose, grounded in evidence and implemented with high fidelity, hold promise to help improve the health of the population.

## Data Availability

Not applicable.
